# Experimental and Numerical Investigation into the Effect of Water Uptake on the Capacitance of an Organic Coating

**DOI:** 10.3390/ma16103623

**Published:** 2023-05-09

**Authors:** Steven A. Policastro, Rachel M. Anderson, Carlos M. Hangarter, Attilio Arcari, Erick B. Iezzi

**Affiliations:** Center for Corrosion Science and Engineering, U.S. Naval Research Laboratory, 4555 Overlook Avenue SW, Washington, DC 20375, USA

**Keywords:** polymer-coated materials, protective applications, EIS, diffusion, water uptake

## Abstract

Water uptake by organic coating systems used for corrosion prevention on airframes is one of the principal contributors to the loss of barrier properties of the coating. We used equivalent circuit analyses of electrochemical impedance spectroscopy (EIS) data to track changes in coating layer capacitance for a two-layer coating system consisting of an epoxy primer and polyurethane topcoat immersed in NaCl solutions with different concentrations and temperatures. The capacitance curve exhibited two different response regions, consistent with the “two-stage kinetics” mechanisms for water uptake by the polymers. We tested several numerical diffusion models of water sorption and found the most successful to be one that varied the diffusion coefficient as a function of polymer type and immersion time and accounted for physical aging processes in the polymer. We employed the Brasher mixing law along with the water sorption model to estimate the coating capacitance as a function of water uptake. The predicted capacitance of the coating was found to be consistent with the capacitance obtained from the EIS data, which is consistent with theories that water uptake occurs via initial rapid transport followed by a much slower aging process. Thus, both these water uptake processes need to be considered when making EIS measurements to assess the state of a coating system.

## 1. Introduction

Atmospheric corrosion is one of the most impactful forms of corrosion because its effects extend from small-scale nuts and bolts to large-scale industrial structures such as heat exchangers, even to infrastructure such as bridges. The cost to control atmospheric corrosion has been estimated to be about 50% of the total cost for all other corrosion prevention measures [1] and coatings form the principal method for controlling this type of corrosion on metallic structures.

Different types of coatings provide corrosion control in different ways. Inorganic coatings, such as Co-Ni alloys [2], Zn-Ni alloys [3], or Al-rich primers [4], provide some protective barrier functions along with cathodic protection of the substrate via preferential dissolution of components of the coating. Organic coatings, including epoxies and polyurethanes, which are of interest to this work, generally provide barrier protection to the substrate [5].

Unfortunately, even though the metallic substrate is protected, coatings undergo their own aging processes once they are exposed to the environment. UV weathering, condensation and salt and pollutant deposition are all examples of aging processes for organic coatings [6]. UV exposure induces photochemical oxidation of surface polymer chains in the coating while the latter processes result in the formation of corrosive electrolytes on the coating surface.

While loss of coating gloss is an indicator of an aging coating, not all coating properties are as easily observable. Thus, other means for determining the state of the coating in relation to its protective properties, besides visual inspection, are employed. One approach, using electrochemical impedance spectroscopy (EIS) measurements, has been used to evaluate organic coatings exposed to corrosive environments [7,8]. EIS is an attractive evaluation technique because a measurement can be made with little disruption to the coating [9]. However, interpretation of EIS data usually involves fitting the data with equivalent circuit models and relating changes in the circuit element parameters to changes in the underlying physical system.

To improve the analysis of EIS data obtained from aging coatings, physical models of the degradation process can provide useful initial estimates to fitting routines or provide bounds on equivalent circuit element parameter values. In equivalent circuit models, coatings are usually represented with a resistor element in parallel with a capacitive element. While the range over which capacitance can change is smaller than the range of resistivities, the change in polymer dielectric properties is sensitive to water uptake. Further, it is the transport of electrolytes and associated ions or dissolved gases through the coating that ultimately leads to the degradation of coating barrier properties or inhibitor exhaustion [10,11]. Thus, the focus of this work is on modeling water uptake in organic polymers found in coating systems.

The prevailing understanding of water uptake by an organic polymer is via a two-stage procedure. During the first stage, there is transport along defects in the coating that occur because of inhomogeneities in polymer cross-linking or other processes that resulted in damage to the coating [12,13]. The second stage occurs via network relaxation processes plasticized by water [14].

In contrast to the proposed first stage process, Li and co-workers [15] examined water uptake in six different epoxy resins using gravimetric analysis, attenuated total reflectance FTIR (ATR-FTIR) measurements and positron annihilation lifetime spectroscopy measurements. They found that water diffusion through the epoxy structure was only weakly correlated with the availability of void space, but the saturation concentration was strongly correlated with the availability of polar sites, such as hydroxyl groups, on the polymer chains.

Boubakri and co-authors [16] identified three regions on sorption curves for water uptake in a thermoplastic polyurethane: a Fickian region, region I, characterized by a linear relationship between the absorption ratio and the square root of the immersion time, a pseudo-Fickian region, region II, where the uptake deviated from the Fickian process and the saturation level depended on the immersion temperature and, lastly, at high immersion temperatures, region III, which had an increasing absorption ratio characterized by the formation of microcracks within the coating that eventually led to mechanical failure.

Huacuja-Sánchez and collaborators [17] also examined aging in polyurethanes using gravimetric measurements and ATR-FTIR spectroscopy. They pointed out that, in the second region of the sorption curve, the deviation from the Fickian region resulted in a lower diffusion coefficient and the diffusion coefficient was further dependent on the saturation. They also showed that the absorption of water by the polymer disrupted H–H bonds between the polymer chains by forming water—polymer H-bonds.

The goal of this work is to develop numerical models of water uptake by an organic polymer coating system that incorporate physical and chemical processes that give rise to the experimentally observed two-stage kinetics of water sorption in both an epoxy primer and polyurethane topcoat. We incorporate the changing physical and chemical processes by continuously varying the diffusivity of the saturation as a function of polymer type, immersion conditions, time and saturation. In order to check the model, we demonstrate that it accurately predicts the measured capacitance of the coating system during immersion in various NaCl solutions.

The remainder of the paper is organized into the following sections:

- An overview of sample preparation and the experimental approach for aging coatings and obtaining EIS data from the aged coatings.
- Equivalent circuit analyses of the EIS data.
- Development and assessment of numerical diffusion models for water uptake by the coating.
- Implementation of a model for the change in polymer dielectric properties in response to water uptake.

## 2. Materials and Methods

### 2.1. Sample Preparation

Bare aluminum alloy 2024-0 panels (Q-Lab Corporation, Westlake, OH, USA), with dimensions of 7.62 cm × 15.24 cm × 0.05 cm, were exposed to a chromic acid anodizing treatment (Almag Plating, Baltimore, MD, USA) per MIL-DTL-81706. This treatment was applied to each side of the panel to thicken the native aluminum oxide and cap the oxide to enhance adhesion between the surface and the primer coating [18].

**Figure 1:**
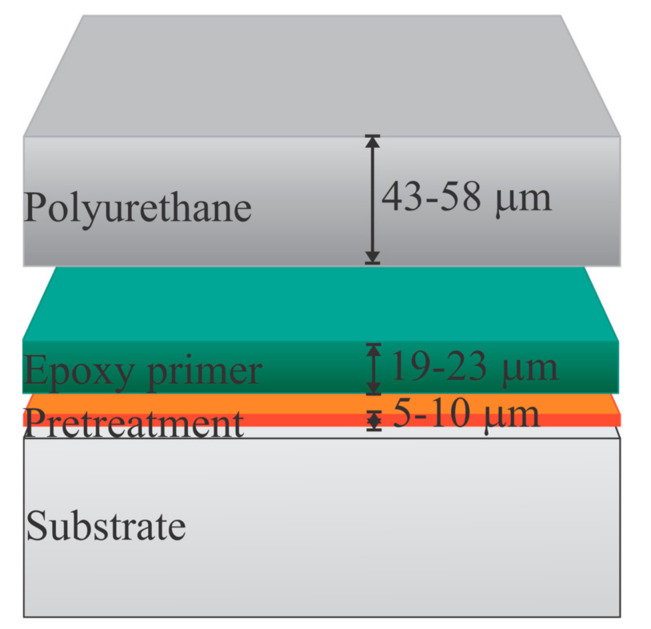
Two coating layers applied to the pretreated substrate.

Panels were then coated with a commercially available aircraft coating system that consisted of a water-reducible epoxy primer, compliant with standard MIL-PRF-85582, Type II, Class C1, barium-chromate-based corrosion inhibitor, (44GN008A; PPG Industries, Pittsburgh, PA, USA) using high-volume, low-pressure (HVLP) spray equipment. After 24 h, dry film thickness (DFT) measurements indicated the primer was 15.24–22.86 μm per manufacturer requirements. Each panel was then coated with a polyurethane topcoat, MIL-PRF-85285, Type IV gray (compliant with standard FED-STD-595 #36375) (99GY003; PPG Industries, Pittsburgh, PA, USA), using HVLP spray equipment. After an overnight cure, DFT measurements indicated the topcoats were within a range of 43.18–58.42 μm per manufacturer requirements, as shown in Figure 1. The coating system was then allowed to further cure in ambient conditions, i.e., 20–22 °C, at 40–60% R.H., for a minimum of 14 d before testing.

Once curing was completed, an electrical attachment was made to each panel and the electrical connection was then sealed using an epoxy adhesive (50112 ClearWeld, J-B Weld, Sulphur Springs, TX, USA).

Electrolyte solutions were mixed using Type I, ultra-pure, ≥18 MΩ-cm water (Milli-Q) and 99% pure, ACS grade, NaCl crystalline powder (Fisher Chemical, Pittsburgh, PA, USA).

Coated coupons, with three replicate coupons per exposure condition, were immersed in one of five conditions:

- Immersion in 0.01 M NaCl at T=5 °C or T=60 °C
- Immersion in 3.2 M NaCl at T=25 °C
- Immersion in saturated NaCl at T=5 °C or T=60 °C

These immersion conditions were selected to simulate the chemistry of an equilibrated thin film electrolyte in atmospheric exposure conditions ranging from 99% to 70% RH in direct sunlight. Test coupons with coatings were placed in glass beakers that were filled with 1.5 L of the specified solution. Beakers with saturated NaCl solutions contained excess salt to ensure saturation. The beakers were covered with mylar sheets to prevent activity changes from evaporative water loss and placed in water baths to maintain the specified temperature. The aging in the NaCl solutions lasted for approximately 3192 h.

### 2.2. EIS Measurements

At specified intervals, each coupon was removed from its respective test environment, washed with Type I, ultra-pure, ≥18 MΩ-cm water (Milli-Q) and dried with laboratory-supplied nitrogen. A cylindrical-bodied, glass paint test cell (PTC1; Gamry, Warminster, PA, USA) was centered and secured to the coupon using a Viton rubber O-ring and a metal clamp. The exposure area for the EIS measurement was 14.6 cm^2^. The cell was filled with 40 mL of 0.6 M NaCl and a 3D printed thermoplastic polymer stopper with ports for a glass-saturated calomel electrode (SCE) reference electrode (Gamry) and a graphite rod counter electrode was used to cap the vessel. The stopper ensured a constant distance between the counter and reference electrodes and the working electrode surface was maintained for each measurement. All measurements were made inside a Faraday cage to minimize external electrical interference.

To make the measurements, a commercial potentiostat (Gamry Interface 1000) was configured to modulate the potential ±10 mV_RMS_ about the system potential. The frequency modulation ranged from 100 kHz to 100 mHz, with frequency values spaced logarithmically, 10 points per decade and averaged over 4 cycles at each frequency. The stationary criterion was met with the ±10 mV_RMS_ perturbation and the linearity of the coupon response to the potential modulation was confirmed using the Kramers—Kronig transform utility provided with the analysis software (Echem Analyst ver. 7; Gamry).

## 3. Results

### 3.1. EIS Data

**Figure 2:**
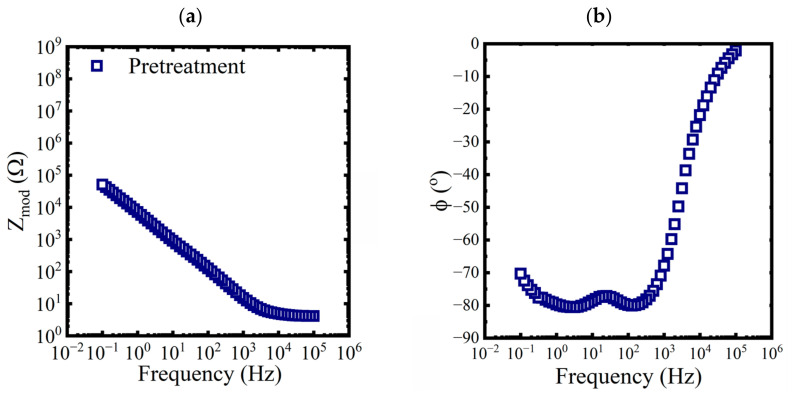
Bode representations of EIS data, (**a**) magnitude and (**b**) phase, for a pretreated panel exposed to a 0.6 M NaCl solution at 25 °C.

Bode diagrams with impedance modulus, $Z_{mod}$, and phase angle as functions of the frequency were used to visualize the spectra. Three replicates of baseline EIS measurements were made on a pretreated test panel in 0.6 M NaCl solution. An example of the data is shown in Figure 2.

**Figure 3:**
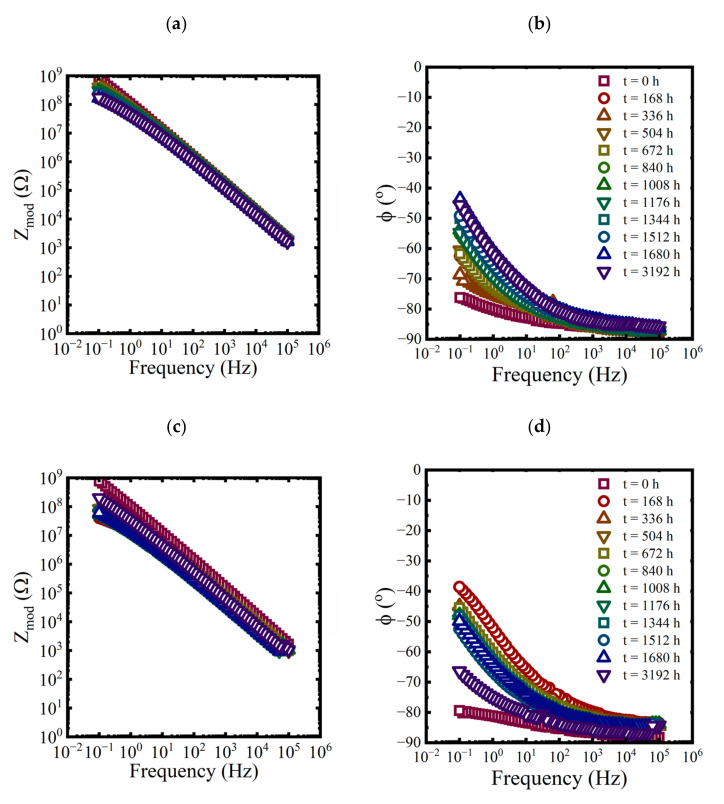
Bode representations of EIS data for a coated panel immersed in a saturated NaCl solution and a 0.01 M NaCl solution at 60 °C for 3192 h. (**a**,**c**) magnitude and (**b**,**d**) phase.

**Figure 4:**
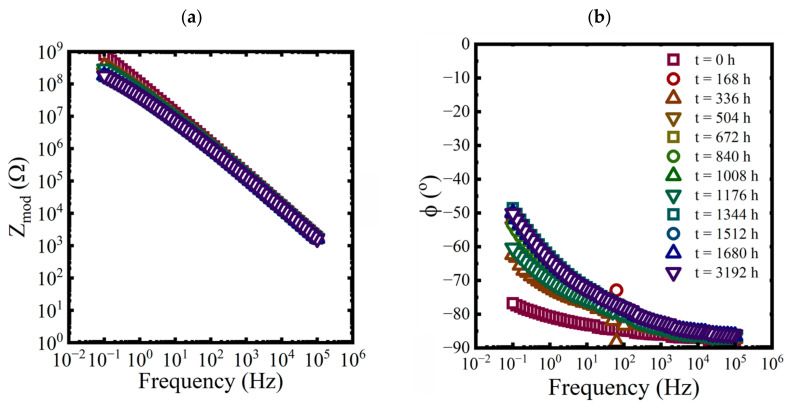
Bode representations of EIS data, (**a**) magnitude and (**b**) phase, for a coated panel immersed in a 3.2 M NaCl solution at 25 °C for 3192 h.

**Figure 5:**
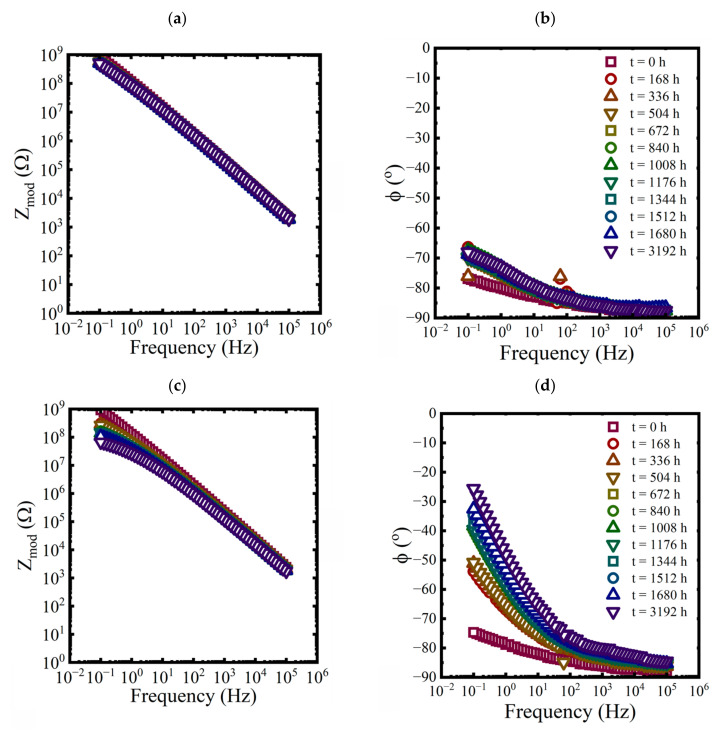
Bode representations of EIS data for a coated panel immersed in a saturated NaCl solution and a 0.01 M NaCl solution at 5 °C for 3192 h. (**a**,**c**) magnitude and (**b**,**d**) phase.

EIS measurements were made on each panel approximately every 168 h for the first 10 weeks and then at 3192 h at the end of the aging period. While three replicate coated panels were aged in each condition, only EIS data from one of the replicates are shown in each plot in Figure 3, Figure 4 and Figure 5.

### 3.2. EIS Analysis

The EIS technique employs a small oscillating excitation signal that is overlaid on a constant potential or current. The amplitude of the excitation signal is kept small to ensure that the system’s response to the excitation will remain nearly linear. The measured response of the system to the excitation signal can then be interpreted to reveal changes to the system’s physical or chemical properties. Analysis of the measured impedance data usually involves fitting an electrical analog model to the data to obtain estimates of the parameters of the circuit components. These parameter values are then used as proxies for the state of the coating.

**Figure 6:**
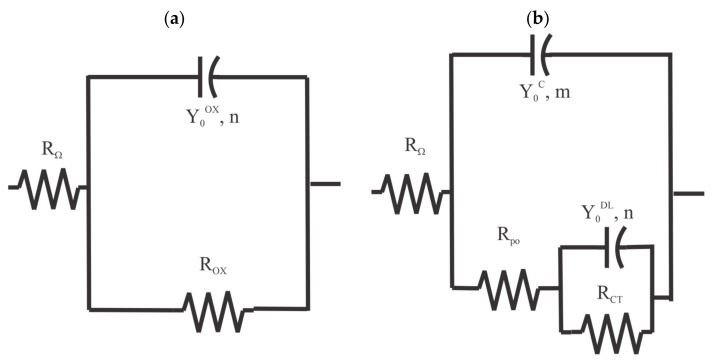
Equivalent circuits used for the EIS analysis. (**a**) two resistors and single CPE circuit. (**b**) three resistors, two CPE circuits.

The EIS spectra for the pretreated samples from Figure 2 were analyzed by fitting them with a single resistor-single constant phase element (CPE) equivalent circuit [19] in series with a solution resistance, as shown in Figure 6a. The EIS spectra for the coated panels were analyzed by fitting them with a two resistor-two constant phase element (CPE) equivalent circuit [20] in series with a solution resistance, as shown in Figure 6b. This type of equivalent circuit is commonly used to model the EIS response of coatings [21]. $R_{\Omega }$ in both circuits indicates the sample-to-reference electrode solution resistance. The behavior of the oxide—electrolyte interface for both the pretreated and coated samples is reflected in the polarization resistance, $R_{CT}$, in parallel with a CPE impedance, $Z_{cpe}^{DL}$. The coating system response is reflected in a pore resistance, $R_{po}$, in parallel with the impedance from a CPE, $Z_{cpe}^{C}$.

For nonreactive elements, the impedance is simply the resistance, R. However, reactive elements generally have real and imaginary impedance contributions. For constant phase elements, the impedance is given by (1) [22],

(1) $$Z_{cpe}\left(\omega \right)=\frac{1}{Y_{0}{\left(j\omega \right)}^{m,n}},$$

where $Y_{0}$ represents a proportionality factor, $j=\sqrt{-1}$, ω is the angular frequency and m, or n, the CPE exponent.

To obtain values of the equivalent circuit element parameters, nonlinear regressions were performed using a simplex algorithm [23,24,25]. Equivalent circuit element parameter values associated with the anodized aluminum oxide are given in Table 1.

These were assumed to be the same values for the analysis of EIS measurements of the coated panels. Note that $R_{\Omega }$ was a constant for all EIS measurements because we consistently used 40 mL of 0.6 M NaCl to fill the PTC and used a 3D printed cap for the PTC that positioned the reference and counter electrodes at a constant height for each measurement.

Representative equivalent circuit element parameter values are given in Table 2 for a single coating system aged in 0.01 M NaCl at T=5 °C for 3192 h. As expected, the pore resistance, $R_{po}$, decreased over the course of the immersion as the coating system aged. The decrease in the CPE exponent, m, indicates the coating is behaving less like an ideal capacitor. This change in the exponent has been associated with changing ionic conductivity [26].

The relationship between $Y_{0}$ and an estimated coating capacitance, $C_{C}$, for values of m near 1, is given in (2),

(2) $$C_{C}=\frac{{\left(Y_{0}^{C}R_{po}\right)}^{\frac{1}{m\;}}}{R_{po}}.$$

**Figure 7:**
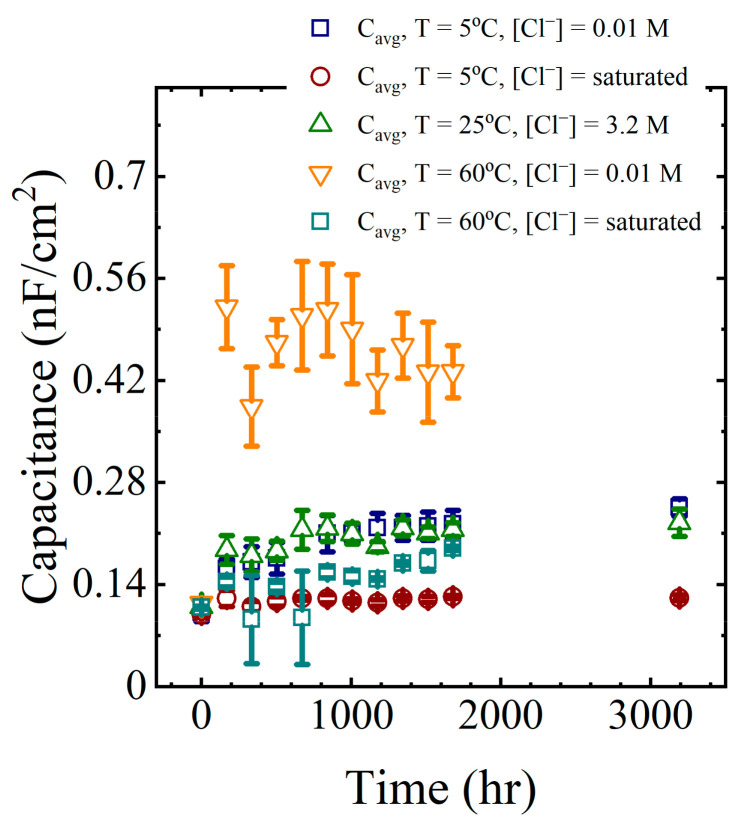
Average coating capacitance values for all aging conditions.

Calculated capacitance values obtained from (2) are shown in the last column of Table 2 for a single panel. The calculation was repeated for all EIS data obtained from all panels exposed to the laboratory aging conditions. These capacitance values are shown in Figure 7 as a function of aging time.

The general trend in the data from all aging conditions indicated a rapid increase in the coating capacitance during the initial few to tens of hours of immersion. This corresponded to the region I sorption, as identified by Boubakri and co-authors [16]. At longer times, the changes in the capacitance appeared to stabilize or increase only slowly. This corresponded to region II sorption [16,17]. The changes in the average capacitance at long times were assumed to be due to water sorption by the epoxy layer.

The saturation function, Ψ, of the coating can be described by (3) [27],

(3) $$\Psi \left(t\right)=\frac{\phi \left(t\right)}{\phi_{s}}=\frac{\ln\left(\frac{C_{c}\left(t\right)}{C_{C}^{0}}\right)}{\ln\left(\frac{C_{c}^{s}}{C_{c}^{0}}\right)}\;,$$

which arises because we assume the capacitance of the coating is a function of lnϵ, the mixed dielectric constant of the water and polymer [28]. The saturation function, which ranges from 0 to 1, is the ratio between the fraction of available free space within the polymer that is filled with water at time, *t*, corresponding to $\phi \left(t\right)$ and the fraction of available free space that is filled with water at saturation, $\phi_{s}$. $C_{c}\left(t\right)$ is the coating capacitance at time, *t*; $C_{c}^{0}$ is the coating capacitance at *t* = 0; and $C_{c}^{s}$ is the coating capacitance at saturation.

## 4. Discussion

### 4.1. Development of Governing Equations for Polymer Saturation

We assume saturation of a polymer coating immersed in solution can be treated as a one-dimensional diffusion process in a plane sheet, subject to the fundamental differential equation of diffusion, as given in (4),

(4) $$\frac{\partial \Psi \left(x,t\right)\;}{\partial t}=\frac{\partial }{\partial x}\left(D\frac{\partial \Psi \left(x,t\right)}{\partial x}\right).$$

The initial condition is given in (5),

(5) $$\Psi \left(x,0\right)=0.$$

A constant Dirichlet boundary condition is assumed at the air—coating interface and a Neumann boundary condition is assumed at the coating—metal interface [29]; both given in (6), respectively,

(6) $$\Psi \left(0,t\right)=\Psi_{s},\;\frac{\partial \Psi }{\partial x}\left(x_{2},t\right)=0,$$

where $\Psi_{s}$ is the saturated volume fraction of the polymer coating occupied by water for the given conditions. $D_{i}$ is the diffusivity of the water in the polymer. x=0 corresponds to the coating—electrolyte interface, $x=x_{1}$ corresponds to the interface between coating layers in later models and $x=x_{2}$ corresponds to the coating—substrate interface. At t=0, there is no water present in the coating.

An analytical solution to the diffusion equation in (4), subject to the initial condition given in (5) and the boundary conditions given in (6), and assuming a constant diffusivity is shown in (7) [30],

(7) $$\Psi \left(x,t\right)=\Psi_{s}-\frac{4\Psi_{s}}{\pi }\overset{\infty }{\underset{n=0}{\sum }}\left(\frac{{\left(-1\right)}^{n}}{2n+1}\right)\left(e^{\frac{-D{\left(2n+1\right)}^{2}\pi^{2}t}{4x_{2}^{2}}}\right)\left(\cos\frac{\left(2n+1\right)\pi x}{2x_{2}}\right).$$

For a plane sheet, the mass fraction of water uptake can be obtained from (8) [30],

(8) $$\Psi \left(t\right)=\frac{M\left(t\right)}{M_{s}}=1-\overset{\infty }{\underset{n=0}{\sum }}\frac{8}{{\left(2n+1\right)}^{2}\pi^{2}}e^{\frac{-D{\left(2n+1\right)}^{2}\pi^{2}t}{4x_{2}^{2}}},$$

where $M\left(t\right)$ is the amount of water uptake after time, *t*; $M_{s}$ is the amount of water uptake needed to saturate the polymer; and Ψ is the saturation function from (3).

The mass fraction of water in the coating is obtained from (9),

(9) $$M\left(t\right)=\rho \phi_{s}\int_{0}^{x_{2}}\Psi \left(x,t\right)dx,$$

where ρ is the density of water.

If unknown, the diffusion coefficient, D, can be estimated by using a Newton—Raphson algorithm to find the roots of (10) at a given time,

(10) $$f\left(t\right)=\frac{M\left(t\right)}{M_{s}}-\frac{\phi \left(t\right)}{\phi_{s}},$$

where the first term is given by (8) and the second term by (3). However, for shorter aging times, (8), can be approximated with (11) [27],

(11) $$\Psi \left(t\right)\;\approx \frac{2}{\sqrt{\pi }}{\left(\frac{D}{x_{2}^{2}}\right)}^{\frac{1}{2}}\;\sqrt{t}.$$

The diffusion coefficient, D, for the initial, region I, water uptake in the polyurethane topcoat was determined from the slope of the linear fit to $\Psi \;\mathrm{vs}\;\sqrt{t}$. The region II diffusion coefficient was calculated as the mean of the diffusion coefficients, obtained from (8), from 200 to 800 h of immersion. Water uptake by the polyurethane topcoat was assumed to be the dominant process over this duration. The diffusivity for water uptake in the epoxy coating was determined as the mean diffusion coefficient over the final two time periods of the coating aging. It was assumed that the topcoat had reached saturation and water uptake by the primer had become the dominant process. These diffusion coefficients are shown in Table 3.

The diffusion coefficient for $D_{polyurethane}^{region\;I}\;$ at T=25 °C in Table 3 is reasonably consistent with values obtained from the literature. That is, diffusion coefficients for water in polyurethane at 25 °C span the range from $5.0\times 10^{-13\;}m^{2}/s$ [31] to $15.6\times 10^{-13\;}m^{2}/s$ [17] so our value was approximately 20% lower. For epoxy, the literature suggests $D_{epoxy}^{region\;I}=3.5\times 10^{-13\;}m^{2}/s$ [32]. Our estimated value for $D_{epoxy}^{region\;I}$ at T=25 °C was $0.8\times 10^{-13\;}{\;m}^{2}/s$, which was roughly 20% lower than the literature value.

### 4.2. Water Sorption Models

Water sorption of the polymer coatings assumes that water molecules occupy adsorption sites along the cross-linked polymer chains and transport occurs via exchange between these sites. The coating systems in the laboratory aging conditions were assumed to be saturated when available adsorption sites were filled. For the numerical models, the polyurethane coating was identified as Layer 1 and the epoxy coating was identified as Layer 2.

#### 4.2.1. Constant Diffusivity Models

These two models were not intended to replicate the water sorption curves. Instead, they were used to provide upper and lower bounds on the coating response. The constant diffusivity models assumed the diffusion coefficients for water uptake in both polymer layers were constant and equal. For these two models, the solution provided by (7) was valid. In the fast water uptake model, $D_{1,2}=0.96\times 10^{-13\;}\;m^{2}/s$. For the slow water uptake model, $D_{1,2}=96\times 10^{-16}\;m^{2}/s$.

#### 4.2.2. Varying Diffusivity as a Function of Depth and Polymer Type

These models were used to develop and test the numerical methods for solving the diffusion equation with a nonconstant diffusivity. They varied the diffusion coefficient as a function of depth, x and polymer. Numerical solutions were obtained using the Crank-Nicolson [33] discretization of the diffusion equation, given in (12),

(12) $$U_{i}^{j+1}-\frac{\nu }{2}\left(U_{i+1}^{j+1}-2U_{i}^{j+1}+U_{i-1}^{j+1}\right)=U_{i}^{j}+\frac{\nu }{2}\left(U_{i+1}^{j}-2U_{i}^{j}+U_{i-1}^{j}\right),$$

where $U_{i}^{j}$ is the discretization of the saturation function, Ψ, at node, *I*, for time-step, *j*. That is, $U_{i}^{j}\approx \Psi \left(x,t\right)\;$ and the time and space variables were nondimensionalized as, $\nu =\delta \tau /{\left(\delta X\right)}^{2}$ with $\tau =Dt/x_{2}{}^{2}$ and $X=x/x_{2}$.

**Figure 8:**
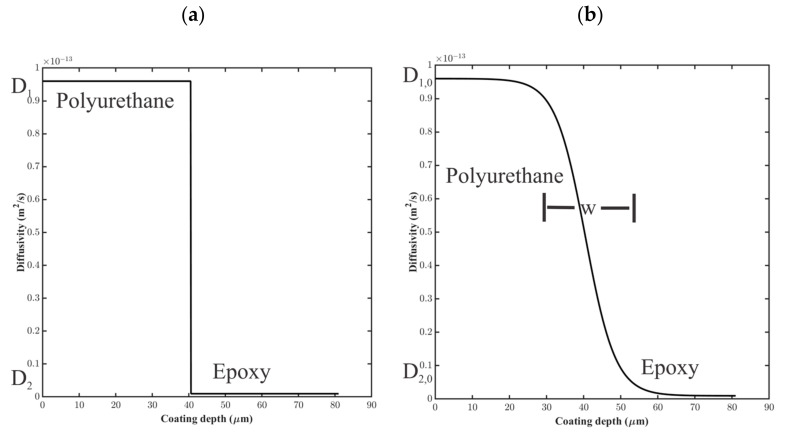
Notional diffusion coefficient profiles for (**a**) step-change model and (**b**) sigmoidal variation model for water uptake in coating layers 1 and 2.

For the step-change model, the diffusion coefficient was assigned using a step function between layers 1 and 2, as shown in Figure 8a, where $D_{1}=0.96\times 10^{-13}\;m^{2}/s$ and $D_{2}=0.8\times 10^{-13}\;m^{2}/s$. At the interfacial point, s,

(13) $$\frac{\partial U_{s,j}}{\partial t}=-\frac{D_{1}}{\delta x}\left(U_{s}-U_{s-1}\right)+\frac{D_{2}}{\delta x}\left(U_{s+1,j}-U_{s,j}\right),$$

which assumed the flux of water leaving Layer 1 was exactly equal to the flux of water entering Layer 2. At the interface, the saturation was assumed to be equal for layers 1 and 2. Numerically solving this equation required modifications to the discretized equation in (12). The modified version is shown in (14),

(14) $$U_{i}^{j+1}-\frac{\nu_{-}}{2}\left(U_{i+1}^{j+1}-U_{i}^{j+1}\right)+\frac{\nu_{+}}{2}\left(U_{i}^{j+1}-U_{i-1}^{j+1}\right)\;\;=U_{i}^{j}+\frac{\nu_{-}}{2}\left(U_{i+1}^{j}-U_{i}^{j}\right)+\frac{\nu_{+}}{2}\left(U_{i}^{j}-U_{i-1}^{j}\right),$$

where, $\tau =\max\left(D\right)t/x_{2}^{2}$, $\nu_{-}=\nu D_{i-1}$ and $\nu_{+}=\nu D_{i+1}$.

In the sigmoidal model, the diffusion coefficient as a function of depth, x, in the coating system was determined using (15),

(15) $$D\left(x\right)=\frac{D_{1,0}-D_{2,0}}{2}\tanh\left(\frac{x}{w}\right)+\frac{D_{1,0}+D_{2,0}}{2},$$

where $D_{1,0}=0.96\times 10^{-13}\;m^{2}/s$ and $D_{2,0}=96\times 10^{-16}{\;m}^{2}/s$, as shown in Figure 8b. The distance, w, small in comparison to the thickness of the coating layers, was the distance over which D varied.

**Figure 9:**
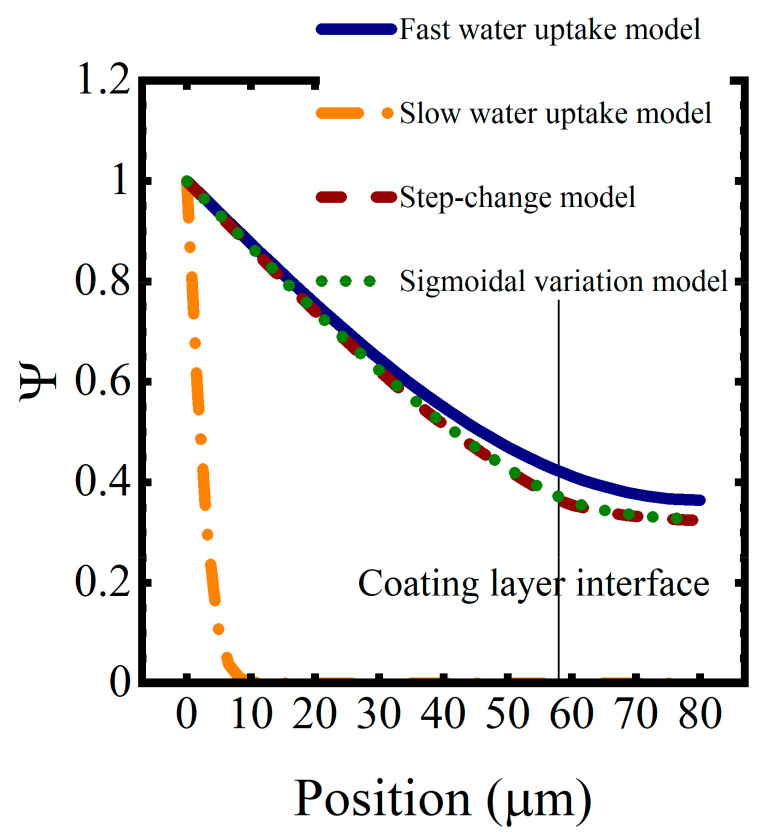
Saturation profiles for the constant and varying-by-polymer-type diffusivity models after one hour of water sorption.

Saturation function profiles resulting from these water uptake models, for a simulated coating system aged in 0.01 M NaCl at T = 5 °C for approximately 1 h, are shown in Figure 9. As expected, coating saturation occurred most quickly in the high diffusivity model and most slowly in the low diffusivity model. The interface between the coating layers disrupted the saturation process, slowing it down because of the lower diffusion coefficients in the epoxy layer. The sigmoidal function for the diffusion coefficient resulted in a slightly faster saturation versus the step change in diffusivity.

Thus, these four simple water uptake models helped establish the numerical framework that was used for the more complex models with variable diffusivities summarized in Table 4 and discussed in the following sections.

#### 4.2.3. Glass-to-Gel Transition Model

This model represents the classic case of diffusion of a liquid into a glassy polymer with a steep concentration gradient that results in an initial water uptake proportional to $\sqrt{t}$ but water uptake behind the concentration gradient is proportional to *t* [34,35,36]. For our system, the diffusion coefficients in both coating layers were treated as functions of polymer type, depth and concentration. Diffusivities were initially low for water transport through the cured, glassy polymer. However, once the saturation surpassed a threshold level, the diffusivity values were then smoothly increased, using a form of (15) to transition the diffusion coefficient from the glass-to-gel value. The threshold saturation corresponded to the transition of the polymer from a glassy state to a gel-like state. The diffusion coefficients for the gel states and glass states for the two polymers are given in Table 4. As a simplification, we assumed the transition between the glassy and gel states was similar for both polymers [37].

The initial uptake of water into the coating occurred as Type I diffusion. This is the classic Fickian diffusion that exhibits a dependence on $\sqrt{t}$. However, once the transition to the gel state was completed, water transport took place as Type II diffusion. In Type II diffusion, the increase in saturation is dependent on *t* [34,38]. The arrow pointing at the near-vertical saturation curve in the saturation profiles shown in the following section indicates the transition between Type I to Type II diffusion.

In this simulation, complete saturation of the coating occurred within a few hours, which was not consistent with the observations of the immersed coating systems, where the capacitance was still changing after 1000–1500 h, as seen in Figure 7. Thus, despite its more complex behavior, the polymer glass-to-gel transition model also did not replicate the observed sorption curves for polymer coatings.

#### 4.2.4. Polymer Aging Model

Possart and Zimmer described three polymer aging mechanisms based on their work on polyurethanes exposed to high relative humidity environments [39]. They suggested that during the initial water uptake, the water molecules separated weak polymer—polymer hydrogen bonds to form stronger polymer—H_2_O hydrogen bonds as well as solvating hydrophilic regions along the polymer chains. This initial phase usually took less than a week to complete. In the second stage of water uptake, dispersed clusters of a mixed phase of water and polymer strands formed throughout the coating. This stage took thousands of hours to complete. They characterized these first two stages as physical aging mechanisms because they were generally reversible by drying out the coating. In the final stage, they observed irreversible chemical aging from the cleavage of the polymer chains via hydrolysis reactions with the water molecules.

In our model, the diffusion coefficients in both coating layers were treated as functions of the polymer type, depth, saturation and immersion time. Water diffusivity was assumed to be high during the initial period of water uptake into the coating, corresponding to the first physical aging stage. Once this initial water uptake was completed, which took roughly seven days, in our immersion experiments, the diffusion coefficients were decreased. That is, once a critical saturation threshold of the polymer was reached, which we assumed to be around 55% for polyurethane and 45–50% for epoxy, the diffusion coefficient was changed from $D_{i}^{initial}$ to $D_{i}\left(t\right)$. This decrease was carried out to account for slower water sorption during the formation of the water-rich and polymer mixed phase.

**Figure 10:**
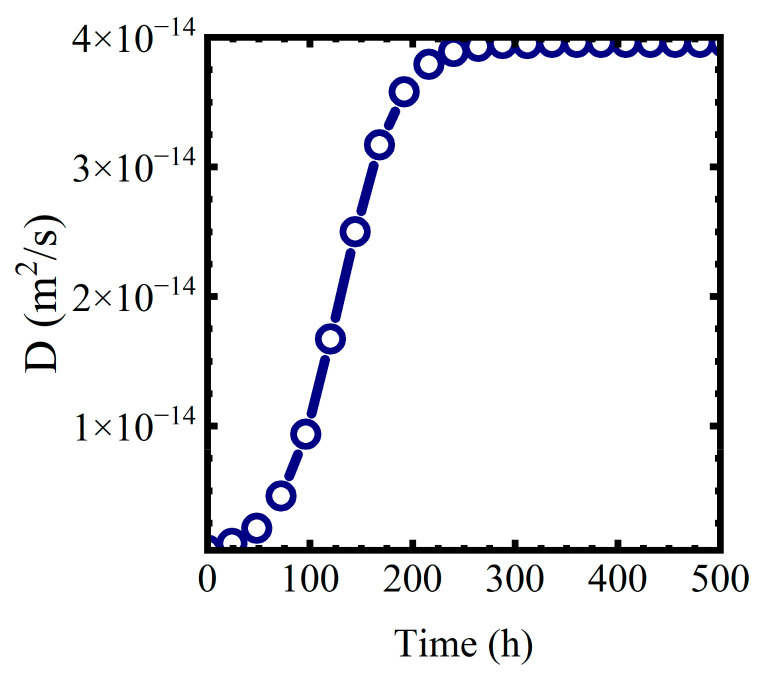
$D_{i}\left(t\right)$ for T=5 °C and $\left[{\mathrm{Cl}}^{-}\right]=0.01\;M$.

An expression to simulate the effect of this aging process on water transport was obtained by solving the logistic equation given in (16),

(16) $$\;\frac{dD_{i}}{dt}=kD_{i}\left(D_{i}^{age}-D_{i}\right),$$

subject to the initial condition, $D_{i}\left(t=0\right)=D_{i,0}$. The subscript, *i*, in (16) differentiates between the polyurethane and epoxy layers. The solution to (16) is given in (17),

(17) $$D_{i}\left(t\right)=\frac{D_{i}^{age}}{\left[\frac{\left(D_{i}^{age}-D_{i,0}\right)}{D_{i,0}}*e^{-D_{i}^{age}\frac{t}{\tau }}\right]+1},$$

where we assume the formation of the mixed phase proceeds as an autocatalytic logistic function [40]. In (17), $D_{i}^{age}$ is the diffusion coefficient at the end of the formation of the mixed water—polymer phase. τ is a characteristic reaction area constant. Values of $\tau \approx 10^{-9}\;m^{2}\;$ worked for this analysis. $D_{i,0}$ represents the diffusion coefficient at the start of the phase formation process. $D_{i,0}$ for both polymers was assumed to be $\approx 0.01\times D_{i}^{age}$. A plot of $D_{i}\left(t\right)$ for T=5 °C and $\left[Cl^{-}\right]=0.01\;M$ is shown in Figure 10.

**Figure 11:**
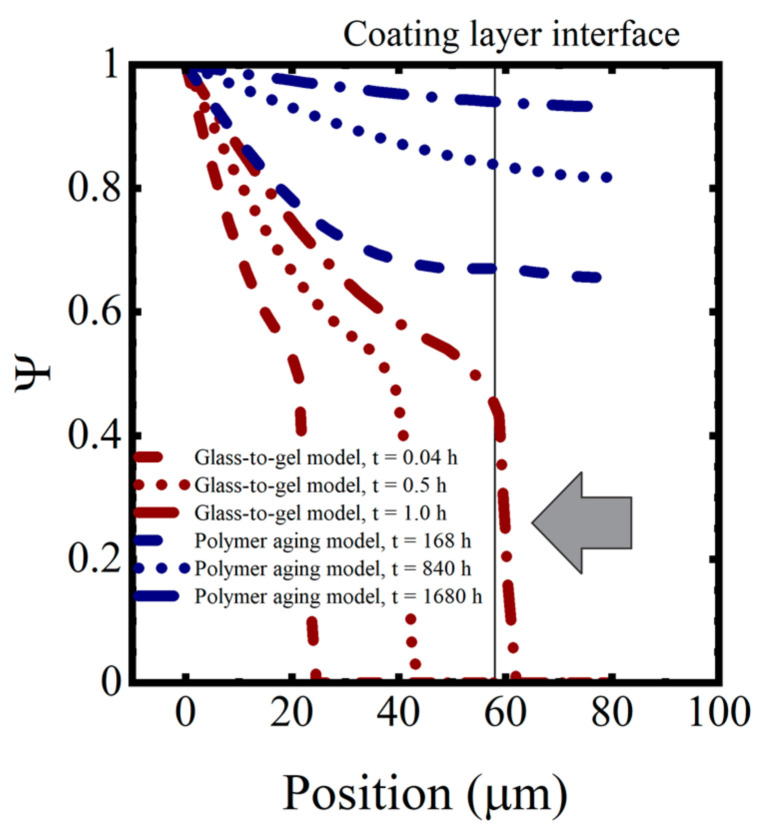
Saturation profiles for the glass-to-gel transition model and polymer aging model.

Numerical values for the various diffusion coefficients in each polymer are given in Table 4. The saturation function profiles resulting from the polymer aging model are shown in Figure 11 for the model coating system aged in 0.01 M NaCl at T = 5 °C. Saturation proceeded much more slowly in this model than in the glass-to-gel transition model, as can be seen by comparing the timestamps on the saturation profiles. The polymer aging model, when coupled with an effective medium theory for changes in the dielectric properties, exhibited the expected behaviors of the water sorption curves for polymers.

### 4.3. Estimating Capacitance from Changes in Dielectric Properties

As was seen in Figure 7, water uptake by the polymer generally increased the capacitance of the coating. The capacitance of a parallel plate capacitor with a dielectric material separating the plates is obtained from (18),

(18) $$C=\frac{\epsilon_{i}\epsilon_{0}A}{d},$$

where $\epsilon_{0}=8.85\times 10^{12}A^{2}s^{4}m^{-3}kg^{-1}$, A is the exposure area, d is the separation between the oppositely charged plates and $\epsilon_{i}$ is the dielectric constant of the material. Values of $\epsilon_{i}$ for materials of interest to this work include water, ϵ=80.1; hydrated Al_2_O_3_, ϵ=11.5 [41]; epoxy, ϵ=3.6  [42]; and polyurethane, ϵ=6.19  [43].

To obtain the capacitance of a parallel plate capacitor in which dielectric properties of the material change throughout its depth, we can envision sectioning the dielectric material into thin slices such that each slice has a constant capacitance. The capacitor is then assumed to behave as a series combination of the individual thin-slice capacitors.

The capacitance of each slice is calculated by assuming the contributions to the dielectric constant of that slice follow the Brasher-mixing law [44]. The total capacitance is then the sum of the individual capacitances, as given in (19),

(19) $$\frac{1}{C_{c}\left(t\right)\;}=\frac{1}{A\epsilon_{0}\epsilon_{polymer}}\sum_{n=1}^{N}\frac{\Delta x}{\exp\left(\left(\Psi_{n}\right)\phi_{s}\;\ln\epsilon_{H_{2}O}\right)},$$

where N is the number of slices of thickness Δx and $\Psi_{n}$ is the saturation function in each slice, n and is obtained from the solution to the diffusion Equation (4).

### 4.4. Predicting Coating Capacitance from the Polymer Aging and Brasher-Mixing Model

Thus, modeling the polymer aging process and evolving coating capacitance consisted of performing the following steps:

- Assign the diffusion coefficient for water sorption in each polymer layer using the constant values provided in the polymer aging entry in Table 4 or from (17), depending on the saturation level. The change in diffusion coefficients across the polymer layer interface was calculated using (15).
- Determine the saturation in both polymer layers by numerical solution of the Crank-Nicolson discretization of the diffusion equation given in (14), subject to the initial condition given in (5), the boundary condition given in (6), and the continuity condition given in (13).
- Calculate the coating capacitance from (19).

**Figure 12:**
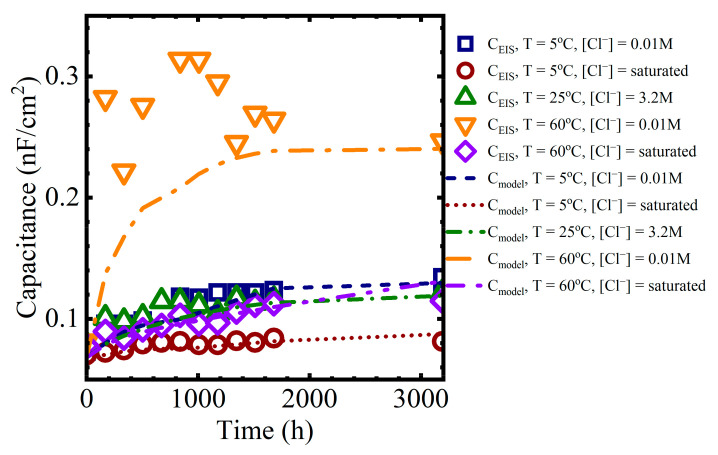
Coating capacitance values obtained from equivalent circuit fits compared to values obtained from the polymer aging and Brasher-mixing model.

The calculated values could then be compared against the capacitances obtained from the EIS data. Example comparisons are shown in Figure 12 for the different immersion conditions:

- Immersion in 0.01 M NaCl at T=5 °C or T=60 °C
- Immersion in 3.2 M NaCl at T=25 °C
- Immersion in saturated NaCl at T=5 °C or T=60 °C

The model predictions for the capacitance were closer to the EIS values for the lower temperature solutions because the experimental data from the solutions at 60 °C had significantly more variability than at lower temperatures.

While there are discrepancies between individual data points and the model values, the overall capacitance trends from the polymer aging and Brasher-mixing model presented here are consistent with the sorption behavior observed in the data. That is, at shorter times, region I of the capacitance curve is a function of Fickian diffusion. At longer times, in region II of the capacitance curve, water sorption occurred as a pseudo-Fickian process, which we suggest was governed by the rate of formation of the water-rich and polymer mixed phase.

These results are consistent with the physical aging mechanisms proposed by Possart and Zimmer [39]. They suggested that irreversible chemical aging of the polymers during water uptake does occur, but that cleavage of the polymer chains themselves is very slow.

These comparisons between model predictions and EIS data indicate that EIS measurements taken at different exposure intervals, over a wide enough frequency range to allow determination of the coating capacitance, can be used to track the saturation of the coating [36,45]. The degree of saturation can then be used to estimate the degree of physical aging in the polymer. We suggest this approach permits a quantifiable measure of the state of the coating, which can then be included as a specific data point in an assessment of the state of the coating to determine if it is still maintaining its protective function. In addition, and independent of any chemical aging processes degrading the polymer chains, saturation of the polymer results in water at the polymer—metal interface, which has been shown to negatively impact coating adhesion to the substrate [46]. Examining and analyzing EIS data to detect water at the polymer—metal interface is a topic for future work.

## 5. Summary and Conclusions

EIS data were obtained from measurements of an organic polymer coating system consisting of a polyurethane topcoat overlaid on epoxy primer that had been applied to pretreated aluminum alloy test coupons that were then immersed in various NaCl solutions at different temperatures for more than 3000 h. A two resistor—two constant phase element (CPE) equivalent circuit model was used to fit the EIS data obtained from the coating systems at approximately 168 h intervals. Parameters from the equivalent circuit model fits were used to estimate changes in the coating capacitance over the course of the immersions.

Numerical solutions of the one-dimensional diffusion equation were developed that used increasingly complex models of the diffusion coefficient for water sorption in the coating system. To explain the features of the water sorption curves for the polymers obtained from the EIS data, we proposed that water uptake by the organic coating system was consistent with the two-stage kinetics mechanism. The first stage, associated with a high diffusivity, resulted in rapid water uptake. In the literature, this mechanism has been associated with the formation of polymer—H_2_O hydrogen bonds following separation of polymer—polymer hydrogen bonds.

The second mechanism was then initiated once the volume fraction of water reached a critical threshold. This mechanism, associated with a varying diffusivity modeled using an autocatalytic logistic function, was consistent with the physical aging mechanism that theorizes further water uptake results in the formation of a water-rich and polymer mixed phase.

Capacitance values predicted from the polymer aging and Brasher-mixing models were consistent with trends observed in the experimental data. These results are important for potential industrial applications because polyurethane and epoxy coatings are widely used in commercial and military aviation and on aluminum materials in industrial applications. This modeling approach allows us to estimate the state of the layers of the coating system using only the type of coating and the exposure conditions, while also providing context for interpreting nondestructive EIS measurements of in-situ coatings.

## Figures and Tables

**Table 1:** Equivalent circuit element parameter values for a pretreated panel.

$R_{\Omega }\;\left(\Omega \right)$	$R_{CT}\;\left(k\Omega \cdot cm^{2}\right)$	$Y_{0}^{DL}\;\left(S\cdot s^{n}\cdot cm^{-2}\right)$	n
4	76	$2.1\times 10^{-9}$	0.895

**Table 2:** Equivalent circuit element parameter values for a coated panel immersed in 0.01 M NaCl at T = 5 °C for 3192 h.

Time (h)	$R_{po}\;\left(G\Omega \cdot cm^{2}\right)$	$Y_{0}^{C}\;\left(S\cdot s^{n}\cdot cm^{-2}\right)$	m	$C_{c}\;\left(nF\cdot cm^{-2}\right)$
0	22.6	$0.08\times 10^{-9}$	0.946	0.09
168	6.13	$0.14\times 10^{-9}$	0.914	0.14
336	5.55	$0.15\times 10^{-9}$	0.912	0.14
504	5.26	$0.15\times 10^{-9}$	0.909	0.15
840	3.07	$0.19\times 10^{-9}$	0.898	0.18
1008	2.63	$0.21\times 10^{-9}$	0.893	0.20
1176	2.77	$0.21\times 10^{-9}$	0.896	0.19
1344	2.63	$0.21\times 10^{-9}$	0.894	0.20
1512	2.34	$0.22\times 10^{-9}$	0.891	0.20
1680	2.04	$0.23\times 10^{-9}$	0.886	0.21
3192	1.31	$0.29\times 10^{-9}$	0.872	0.25

**Table 3:** Estimated diffusion coefficients for polyurethane and epoxy from Figure 7.

T (°C)	$\left[Cl^{-}\right]\;\left(M\right)$	$D_{polyurethane}^{region\;I}\;\left(\frac{m^{2}}{s}\right)$	$D_{polyurethane}^{region\;II}\;\left(\frac{m^{2}}{s}\right)$	$D_{epoxy}^{region\;II}\;\left(\frac{m^{2}}{s}\right)$
5	0.01	$0.41\times 10^{-13}$	$0.40\times 10^{-13}$	$0.23\times 10^{-13}$
5	Saturated	$0.07\times 10^{-13}$	$0.05\times 10^{-13}$	$0.25\times 10^{-13}$
25	3.20	$0.96\times 10^{-13}$	$0.76\times 10^{-13}$	$0.31\times 10^{-13}$
60	0.01	$4.0\times 10^{-13}$	$1.3\times 10^{-13}$	$0.35\times 10^{-13}$
60	Saturated	$2.3\times 10^{-13}$	$2.3\times 10^{-13}$	$0.19\times 10^{-13}$

**Table 4:** Complex variable diffusivity water sorption models.

Model	Summary	$D\;\left(\frac{m^{2}}{s}\right)$
Glass-to-gel transition model	*D* varies as a function of position in the coating and composition. Glass-to-gel transition of the polymer.	$D_{1}^{glass}=96\times 10^{-16\;}$ $D_{1}^{gel}=0.96\times 10^{-13\;}$
		$D_{2}^{glass}=80\times 10^{-16\;}$ $D_{2}^{gel}=0.8\times 10^{-13\;}$
Polymer aging model	*D* varies as a function of position and time. Rapid uptake of water into the polymer followed by a slow polymer relaxation.	$D_{1}^{initial}=0.96\times 10^{-13\;}$ $D_{1}^{age}=0.76\times 10^{-13}$ $D_{1,0}=0.01*D_{1}^{age}$
		$D_{2}^{void}=0.8\times 10^{-13\;}$ $D_{2}^{age}=0.31\times 10^{-13\;}$ $D_{2,0}=0.01*D_{2}^{age}$

## Data Availability

The data presented in this study are available on request from the corresponding author. The data are not publicly available due to their use in ongoing analysis for another study.
